# Alterations in Biochemical Characteristics, Flavor, and Microbial Community During the Storage of Suancai

**DOI:** 10.3390/foods14203490

**Published:** 2025-10-14

**Authors:** Xingyun Xie, Zehui Li, Hong Liu, Huayi Suo, Zsolt Zalán, Jiajia Song, Yu Zhang

**Affiliations:** 1 College of Food Science, Southwest University, Chongqing 400715, Chinahui1108@email.swu.edu.cn (Z.L.);; 2 National Teaching Demonstration Center of Food Science and Engineering, Southwest University, Chongqing 400700, China; 3 Yibin Academy of Southwest University, Yibin 644000, China; 4 The Agricultural and Rural Affairs Bureau of Pingshan, Yibin 645350, China; 5 Food Science and Technology Institute, Hungarian University of Agriculture and Life Sciences, Buda Campus, Herman Ottó Str. 15, 1022 Budapest, Hungary; 6 Chinese-Hungarian Cooperative Research Centre for Food Science, Chongqing 400715, China

**Keywords:** Suancai, non-volatile metabolites, flavor compounds, microbial community composition

## Abstract

This study examined how storage influences the flavor profile and microbial composition of Suancai. To achieve this, we combined physicochemical measurements with analyses of both volatile and non-volatile metabolites, assessments of microbial diversity, and multivariate statistical modeling. Specifically, we investigated changes in physicochemical parameters, organic acids, amino acids, biogenic amines, volatile compounds, and microbial communities before and after storage. The results revealed that, after one year of storage, the pH and nitrite levels in Suancai decreased significantly, whereas the titratable acidity increased substantially. Among the organic acids, lactic acid and oxalic acid remained dominant both before and after storage. In terms of amino acid composition, glutamic acid, aspartic acid, and alanine were present in relatively high concentrations. A total of 69 volatile compounds were detected in *Suancai*, with esters and alcohols representing the major groups. Odor activity value analysis identified nonanal and phenethylaldehyde as key contributors to the overall aroma profile. Correlation analysis further indicated that *Lactobacillus*, *Halomonas*, and *Chromohalobacter* were the dominant bacterial genera linked to flavor development. Specifically, *Lactobacillus* exhibited strong positive correlations with phenylethanal, nonanal, ethyl octanoate, and ethyl decanoate, whereas *Debaryomyces* was positively associated with allyl isothiocyanate, 3-butenyl isothiocyanate, and ethyl ionone.

## 1. Introduction

Suancai is a traditional Chinese fermented vegetable dish with a long history and distinct regional variations, noted for its pronounced fermented aroma and characteristic sour-crisp texture. In northern China, where long, harsh winters make fresh vegetable preservation challenging, Chinese cabbage is commonly processed into Suancai through salting and fermentation driven by lactic acid bacteria [1]. In southern China, the humid climate favors the use of vegetables such as Chinese cabbage and mustard greens, often combined with chili peppers and various spices, to produce Suancai with a more complex and robust flavor profile [2]. Beyond its distinctive taste, Suancai is also valued for its nutritional richness, providing minerals, vitamins, flavonoids, and dietary fiber. In addition to meeting basic dietary needs, it has been reported to confer several health benefits, including modulation of gut microbiota, cholesterol reduction, antioxidant activity, and even potential anti-cancer properties [3,4].

Flavor is a key quality attribute of fermented vegetables, as it directly affects consumer acceptance and is closely tied to microbial community dynamics and metabolic activity [5,6]. Previous studies on industrially produced paocai have examined bacterial and fungal succession alongside flavor development during 365 days of fermentation. These investigations revealed that bacteria exert a much greater influence on flavor formation than fungi, with *Halanaerobium* and *Halomonas* showing particularly strong associations with flavor development. It is noteworthy that *Halanaerobium* and *Halomonas* showed significant negative correlations with allyl isothiocyanate, 3-butenyl isothiocyanate, and 2-phenylethyl isothiocyanate. The reduction in these isothiocyanates led to a weakening of the pungent odor in the fermented vegetables [7]. Li et al. investigated microbial community dynamics and changes in volatile organic compounds during bamboo shoot fermentation in Guangxi and found that *Lactobacillus*, *Paenalcaligenes*, *Kazachstania*, and *Geotrichum* were strongly associated with the formation of key flavor compounds. *Lactobacillus* metabolizes lactate and acetate, which imparts the characteristic sour taste to fermented vegetables. Furthermore, during vegetable fermentation, these acids can interact with alcohols, aldehydes, and ketones to generate a variety of novel flavor compounds, thereby contributing to the product’s complex flavor profile [8]. More broadly, microbial communities play a central role in regulating flavor development in fermented vegetables. For instance, co-fermentation of cabbage with *Lactobacillus plantarum* and *Rhodotorula mucilaginosa* has been shown to enhance desirable flavor compounds such as lactic acid and acetoin, while simultaneously reducing the presence of bitter-tasting amino acids [9]. Yang et al. demonstrated that inoculating *Lactiplantibacillus plantarum* during radish paocai fermentation not only enhanced the diversity and abundance of volatile compounds but also improved product safety by lowering pH and reducing nitrite levels [10]. Currently, numerous studies focus on the correlations between microbial communities and flavor attributes during vegetable fermentation. However, there is insufficient attention paid to the storage phase, a critical period determining the final product quality. There is a particular lack of systematic analysis comparing the dynamic changes in non-volatile metabolites (e.g., organic acids and amino acids), volatile flavor compounds, and microbial communities in Suancai before and after storage. To address this gap, this study established a one-year storage period, a design that aligns with the practical lifecycle of industrial Suancai from production and packaging to distribution and final consumption, where the product shelf life typically spans several months to a full year. This research gap limits the systematic understanding of the mechanisms underlying Suancai quality formation and preservation and also hinders the advancement of quality control technologies in industrial production. Therefore, elucidating the changes and interrelationships among microorganisms and metabolites in Suancai before and after storage is of great importance for enhancing product quality and optimizing production processes.

This study examined Suancai both at the end of fermentation and after one year of storage. Analyses included physicochemical characterization, profiling of volatile and non-volatile metabolites, and assessment of microbial community composition and dynamics using high-throughput sequencing. To gain an integrated understanding, multiple multivariate statistical approaches were applied to evaluate the impact of storage on product quality and to investigate associations between metabolites and microbiota. The findings provide new insights into microbiota–flavor interactions in Suancai before and after storage. This study will provide key scientific data for developing new strategies to predict product shelf life and formulating precise storage management protocols, ultimately facilitating the standardization and enhanced control within the fermented vegetable industry.

## 2. Materials and Methods

### 2.1. Preparation of Suancai

The mustard greens (*Brassica juncea* (L.) Czern.) used in this study were harvested at the end of their vegetative growth stage. The entire aerial parts of the plant, including leaves, petioles, and the central stem, were utilized.

The fermentation process was carried out in a 15 L traditional ceramic jar. The total amount of salt used was calculated as 5% of the initial fresh weight of the mustard greens. A specific layering and salting procedure was adopted: a base layer of whole mustard greens (approximately 10–15 cm thick) was tightly arranged at the bottom of the jar and sprinkled with 20% of the total salt. A second layer of vegetables of similar thickness was then added, followed by the application of 30% of the salt. Finally, a third layer was placed on top and covered with the remaining 50% of the salt.

The initial brine was not pre-formulated but generated in situ through dry-salting, whereby the applied salt drew moisture from the vegetables via osmosis. Salt concentration was supplemented to 8 g/100 g after 5 days and to 16 g/100 g after 30 days. These supplements were achieved by adding dry salt directly into the fermentation tank in amounts calculated as a fixed percentage of the initial vegetable weight. To ensure uniform salt distribution and avoid localized high concentrations, the brine was periodically circulated using a sterilized pump at set intervals (e.g., every 10 days during the first 30 days).

Upon completion of the 180-day fermentation period, the salt concentration was increased to 18 g/100 g, after which a portion of the Suancai was transferred to a dark environment maintained at 18 °C for one year of long-term storage. To maintain the chemical and microbial integrity of all analytical samples throughout the extended testing period, a standardized preservation protocol was implemented. Samples collected at the end of fermentation and after one year of storage were immediately vacuum-packaged to remove oxygen and then stored uniformly at −18 °C until analysis.

Samples of Suancai fermented for 180 days (SF180: Suancai fermented for 180 days) and 1-year-aged Suancai post-fermentation (SP1Y: 1-year-aged Suancai post-fermentation) were provided by Fuling Zhacai Group Co., Ltd. (Chongqing, China).

### 2.2. Determination of Physicochemical Properties

The pH and salinity of Suancai samples were measured by using a PHS-320 meter (Fangzhou Technology, Chengdu, China) and a salinity meter (DELIXI, Zhejiang, China), respectively. Titratable acidity was determined by using the acid–base titration method according to the Chinese National Standard GB 12456-2021 [11], with results expressed as lactic acid. The nitrite content was determined in accordance with the Chinese National Standard GB 5009.33-2016 [12]. Specifically, 5.0 g of Suancai homogenate was extracted with hot water assisted by a saturated borax solution. Proteins were precipitated with potassium ferrocyanide and zinc acetate. The resulting filtrate was then subjected to a diazo-coupling reaction with sulfanilic acid and naphthylethylenediamine dihydrochloride, forming a purplish-red azo dye. After 15 min of color development, the absorbance was measured at 538 nm for the quantification of nitrite.

### 2.3. Determination of Non-Volatile Compounds

#### 2.3.1. Organic Acids

The determination of organic acids was performed according to a previously reported method with minor modifications [13]. Determination was performed using an HPLC system equipped with an ultraviolet detector (Ultimate 3000, Thermo Fisher Scientific, Waltham, MA, USA). Then, 25 g of the sample was homogenized with an equal volume of water, and 5 g of the homogenate was mixed with 15 mL of 80% ethanol solution, incubated in a 75 °C water bath for 0.5 h, cooled to room temperature, and adjusted to a final volume of 25 mL. The mixture was centrifuged, and the supernatant was filtered through a 0.22-µm membrane for subsequent analyses.

C18 column (250 mm × 4.6 mm, 5 μm, Agilent Technologies, Santa Clara, CA, USA), column temperature: 30 °C. The mobile phase consisted of 0.05 mol/L potassium dihydrogen phosphate (pH 2.8) as mobile phase A and 100% methanol as mobile phase B. An isocratic elution program was employed with a constant mobile phase ratio of 97:3 (*v*/*v*, A:B). The total run time was 18 min. Flow rate: 0.8 mL/min, UV detection wavelength: 210 nm, injection volume: 10 μL.

#### 2.3.2. Free Amino Acids

The determination of free amino acids was conducted according to Tang’s method, with minor modifications [14]. 1 g of the dried and ground sample was dissolved in 0.1 mol/L hydrochloric acid, subjected to ultrasonic extraction for 40 min, diluted to 25 mL, and then filtered. Then, take 2 mL of the supernatant and derivatize it with phenyl isothiocyanate and triethylamine. Filter the derivatized lower-layer solution through a 0.22-μm membrane filter and proceed with the instrumental analysis.

Analysis was conducted using a high-performance liquid chromatography (HPLC) system (LC-20A, Shimadzu Corporation, Kyoto, Japan) using C18 column (250 mm × 4.6 mm, 5 μm), column temperature: 35 °C, mobile phase A: 0.05 mol/L sodium acetate solution (pH 6.5), mobile phase B: methanol–acetonitrile–water (2:6:2, *v*/*v*), flow rate: 0.8 mL/min, UV detection wavelength: 254 nm, injection volume: 20 μL. The elution program was as follows: 0 min: 5% B, 7 min: 65% B, 39 min: 49% B, 40–50 min: 100% B, 51–60 min: 5% B.

#### 2.3.3. Biogenic Amines

The determination of biogenic amines was performed using high-performance liquid chromatography, following the method described by Zang et al. [15], with minor modifications. Take 10 g of the sample, 16 mL of 5% trichloroacetic acid, and 2 mL of the internal standard solution (1 mg/mL 1, 7-diaminoheptane) were added, and the solution was ultrasonicated for 1 h, and centrifuged at 2800× *g* for 8 min. The supernatant was removed and diluted to 20 mL with 5% trichloroacetic acid. From this, 2 mL of the solution was derivatized with sodium chloride solution, sodium hydroxide solution, sodium bicarbonate solution, and dansyl chloride solution for 15 min, treated with 200 µL of ammonia solution to terminate the reaction, after which the upper layer was collected, filtered through a 0.22-µm membrane filter, and subjected to detection analysis.

C18 column (250 × 4.6 mm, 5 μm), column temperature 25 °C. Mobile phase A: ultrapure water; mobile phase B: acetonitrile; UV detection wavelength 254 nm; flow rate 1 mL/min; injection volume 20 μL.

#### 2.3.4. Taste Activity Value

The Taste Activity Value (TAV) was used to quantify the flavor contribution intensity of the taste substances. Compounds with a TAV of less than 1 are generally considered to impart no taste contribution in a food matrix. Conversely, a TAV greater than 1 indicates that the compound makes a significant contribution to the overall flavor profile and is classified as a taste-active compound. The intensity of this contribution increases with higher TAV values. TAV was calculated using the following formula: TAV = C_nv_/T_nv_ [16]. C_nv_ represents the concentration of the taste substance (mg/100 g), and T_nv_ is the taste threshold of the corresponding substance (mg/100 g). The taste thresholds were obtained from previous studies [17].

### 2.4. Determination of Volatile Compounds

Volatile compounds were determined using headspace solid-phase micro-extraction gas chromatography-mass spectrometry (HS-SPME/GC-MS) as per a previous study with minor modifications [18]. A 3 g strip sample (1 cm) was placed into a headspace vial to which 5 μL methyl heptanoate (internal standard, 17.58 g/L) was added. The vial was sealed and incubated in a 75 °C water bath for 45 min. Headspace injection was performed with 20 min extraction and 5 min desorption.

Determination was performed on the GC-MS-QP2010 system (Shimadzu Corporation) equipped with a DB-5MS UI capillary column (30 m × 0.25 mm × 0.25 μm). Helium was used as the carrier gas at a flow rate of 1.0 mL/min, with splitless injection and an injector temperature of 220 °C. The initial temperature was 35 °C, which was maintained for 4 min, raised to 110 °C at 10 °C/min, and held for 6 min, followed by an increase to 150 °C at 5 °C/min and held for 2 min, with a final elevation to 230 °C at 7 °C/min and maintained for 6 min. The MS conditions were ion source 230 °C, EI source 150 °C, scan range 50–550 *m*/*z*. The identification of each compound was performed by comparing the mass spectrometry data of volatile compounds with the NIST14 database, and the semi-quantitative concentration of volatile compounds was determined by the ratio of their peak areas to that of the internal standard.

The odor activity value (OAV) was calculated as: OAV = C/OT, where C is the concentration of volatile flavor compounds and OT is the odor threshold. This parameter reflects the contribution of volatile substances to the overall flavor of samples [19].

### 2.5. DNA Extraction and Sequence Analysis of Microorganisms

#### 2.5.1. DNA Extraction of Microorganisms

Genomic DNA was extracted and detected using 1% agarose gel electrophoresis. The bacterial 16S rRNA gene was amplified with primers 799F/1193R, whereas the fungal internal transcribed spacer (ITS) region was amplified via PCR with primers ITS1F/ITS2R (PCR instrument: ABI GeneAmp^®^ 9700, Thermo Fisher Scientific, Waltham, MA, USA). The PCR products of the same sample were mixed and detected by 2% agarose gel electrophoresis. The PCR products were recovered by gel cutting using the AxyPrepDNA Gel Recovery Kit (AXYGEN Company, Union City, CA, USA), eluted with Tris-HCl, and detected by 2% agarose electrophoresis. Based on the preliminary quantification by electrophoresis, the PCR products were quantified using the QuantiFluor™-ST Blue Fluorescence Quantification System (Promega, Madison, WI, USA) and then mixed in the corresponding proportions according to each sample’s sequencing quantity requirements.

#### 2.5.2. Sequence Data Analysis

A paired-end sequencing strategy (2 × 300 bp) was employed. A total of 640,230 bacterial raw reads and 422,117 fungal raw reads were obtained. After sample splitting of the PE reads obtained by sequencing, the double-ended Reads were first subjected to quality control and filtering based on the sequencing quality. Meanwhile, they are spliced according to the overlap relationship between the double-ended reads to obtain the optimized data after quality control splicing. The optimized data were processed by using the sequence denoising methods (DADA2/Deblur) to obtain amplicon sequence variant (ASV) representative sequences and abundance information [20]. Ultimately, 405,222 bacterial clean reads and 413,084 fungal clean reads were obtained. Taxonomic annotation of bacterial 16S rRNA gene ASVs was performed using the SILVA 138 database with a confidence threshold set at 70%. For fungal ITS ASVs, annotation was conducted using the UNITE 9.0 database. Based on the ASV representative sequences and their abundance information, a series of statistical analyses can be conducted, including taxonomic classification analysis, community diversity analysis, species differential analysis, and correlation analysis. The sequencing data have been deposited in the NCBI Sequence Read Archive (SRA) database. The BioProject accession number was PRJNA1335406. The sequencing service was provided by Shanghai Majorbio Bio-pharm Technology Co., Ltd., Shanghai, China.

### 2.6. Data Statistical Analysis

An independent samples *t*-test was conducted using SPSS 27.0 software (SPSS Inc., Chicago, IL, USA). *p* < 0.05 was considered to indicate statistical significance. All measurements are expressed as the mean ± standard deviation (SD). It is noted that the amino acid content is reported on a dry weight basis, while all other indicators are reported on a fresh weight basis. Column charts, stacked plots, and Spearman’s correlation analysis were generated using Origin 2021 (OriginLab Corporation, Northampton, MA, USA). Orthogonal partial least squares-discriminant analysis (OPLS-DA) was conducted using SIMCA 14.1 (Umetrics, Malmo, Sweden). Heatmaps and cluster analysis were created by https://www.chiplot.online/ (accessed on 29 April 2025). All experiments were performed in triplicate.

## 3. Results

### 3.1. Analysis of Physicochemical Properties

The variations in pH, titratable acidity, salinity, and nitrite content of the two Suancai samples are summarized in Table 1. Compared with samples fermented for 180 days, those stored for one year exhibited a significantly lower pH and markedly higher titratable acidity. A low pH environment not only influences the sensory attributes of fermented foods but also plays a critical role in suppressing spoilage and pathogenic microorganisms, thereby contributing simultaneously to product quality and food safety [21]. The nitrite content decreased from (0.67 ± 0.00) mg/kg at 180 days of fermentation to (0.09 ± 0.00) mg/kg after one year of storage. Both values were well below the maximum permissible limit of 20 mg/kg for pickled vegetables established in the Chinese food safety standard (GB 2762-2022 [22]). In fermented foods, nitrite concentration is a critical safety indicator, as it is generated through the metabolic activity of nitrate-reducing bacteria [23]. Therefore, controlling nitrite content is essential for ensuring the safety of Suancai. Thus, controlling nitrite levels is essential for ensuring the safety of Suancai. The results of this study demonstrate that storage markedly decreases nitrite content, thereby improving product safety. This reduction can be largely attributed to the sustained growth of lactic acid bacteria during storage, which lowers the pH and creates an acidic environment unfavorable for nitrate-reducing bacteria. As a result, the pathways responsible for nitrite formation are effectively suppressed, leading to significantly reduced nitrite accumulation [24].

### 3.2. Analysis of Non-Volatile Compounds

#### 3.2.1. Organic Acids

**Figure 1:**
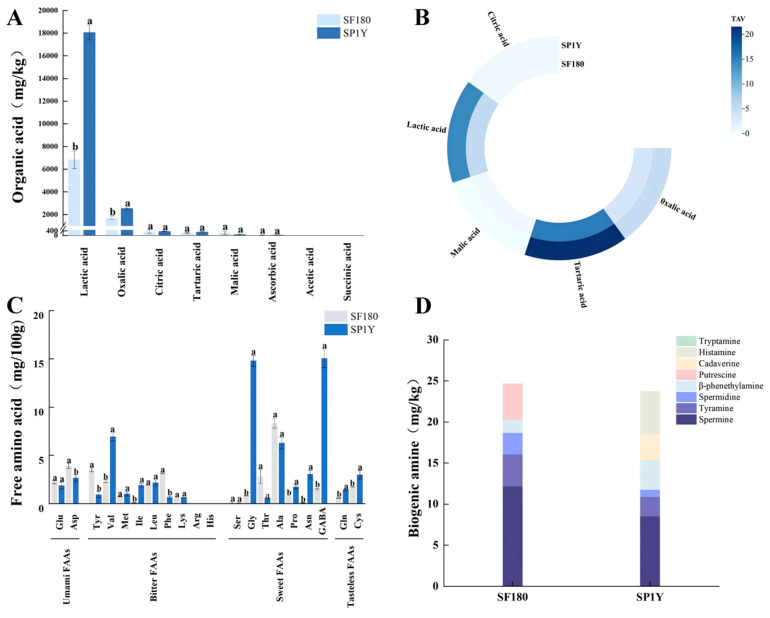
(**A**) Organic acids. Different lowercase letters indicate differences in different Suancai (*p *< 0.05); (**B**) TAV of organic acids; (**C**) Free amino acids. Different lowercase letters indicate differences in different Suancai (*p *< 0.05); (**D**) Biogenic amines. SF180: Suancai fermented for 180 days; SP1Y: 1-year-aged Suancai post-fermentation. All data are presented on a fresh weight basis, except for the free amino acid content, which is reported on a dry weight basis.

The organic acid profiles of the two Suancai samples are presented in Figure 1A. Organic acid composition is an important indicator of microbial activity during fermentation and plays a crucial role in determining the final quality of fermented products [25]. Six types of organic acids were detected in both samples. The total organic acid content was significantly higher in Suancai stored for one year (21469.78 ± 824.19 mg/kg) compared with that in samples fermented for 180 days (9341.70 ± 538.81 mg/kg) (*p* < 0.01). In the Suancai samples analyzed in this study, lactic acid and oxalic acid were identified as the predominant organic acids, whereas tartaric, malic, citric, and ascorbic acids remained at relatively low concentrations. Previous research on the fermentation of vegetables such as mustard greens and radish has shown a similar trend, with lactic acid gradually accumulating during fermentation while the levels of other organic acids decline [26]. During fermentation, citric acid is metabolized into oxaloacetate, which is further converted by transaminase into aspartic acid [27]. As a result, citric acid levels remained low in both Suancai samples. Similarly, malic acid, an intermediate of the tricarboxylic acid (TCA) cycle, is consumed in the later stages of fermentation, accounting for its low concentration. By contrast, oxalic acid and lactic acid accumulated markedly during storage. After one year, the oxalic acid content reached 2553.30 ± 64.29 mg/kg, while lactic acid increased to 18,086.70 ± 655.77 mg/kg, both showing significant increases (*p* < 0.05) compared with levels in Suancai fermented for 180 days. The most notable change was observed in lactic acid concentration, which was more than twice as high in Suancai stored for one year compared with samples fermented for 180 days. Consistently, the one-year stored samples also displayed the lowest pH values. These findings suggest that lactic acid bacteria remain metabolically active during storage, continuing to utilize available substrates and thereby driving ongoing lactic acid production.

To further assess the contribution of different organic acids to the flavor profile of Suancai, their TAVs were calculated, as shown in Figure 1B. Within this evaluation framework, a compound is considered taste-active when its TAV exceeds 1, with higher TAV values reflecting a greater impact on the overall taste characteristics of Suancai [28]. The analysis identified tartaric acid, lactic acid, and oxalic acid as the taste-active organic acids in both types of Suancai, playing a significant role in shaping their flavor profiles. This observation is consistent with the findings of Xiong et al. [29]. Previous studies have further highlighted the role of lactic acid in softening the overall taste of pickled cabbage by offsetting the effects of stronger, more astringent acids, while simultaneously enhancing the microbiological stability of fermented products [30]. The TAV values of tartaric acid were 15.33 in Suancai fermented for 180 days and 21.55 in samples stored for one year. Despite its relatively low concentration, tartaric acid exhibited the highest TAV values among all detected organic acids, indicating its dominant contribution to the overall taste profile.

#### 3.2.2. Free Amino Acids

As presented in Figure 1C, a total of 18 free amino acids were detected in both types of Suancai. Non-volatile, free amino acids can be directly perceived by taste receptors, contributing to fundamental flavor attributes such as umami, sweetness, and bitterness [31]. More importantly, they act as crucial precursors for the synthesis of diverse flavor compounds, thereby enriching the overall flavor complexity and enhancing the sensory appeal of the product [32]. Overall, sweet amino acids were the most abundant in both Suancai samples. In Suancai stored for one year, the concentration of sweet amino acids was approximately three times higher than that of bitter amino acids and nine times higher than that of umami amino acids. In the 180-day fermented samples, alanine (8.34 ± 0.51 mg/100 g) was the predominant free amino acid, followed by aspartic acid, threonine, and phenylalanine. In contrast, Suancai stored for one year was dominated by γ-aminobutyric acid (GABA) (15.07 ± 0.96 mg/100 g) and glycine (14.82 ± 0.57 mg/100 g), followed by alanine and valine. These results indicate that prolonged storage significantly alters the free amino acid profile of Suancai. GABA, a non-protein amino acid produced via the decarboxylation of L-glutamic acid by the enzyme glutamate decarboxylase (GAD), has attracted considerable interest due to its reported physiological functions, including alleviation of insomnia and depressive symptoms, enhancement of immune activity, and regulation of blood pressure [33]. One year of storage led to an approximately tenfold increase in γ-aminobutyric acid content compared with Suancai fermented for 180 days, indicating that post-fermentation storage may play an important role in promoting GABA biosynthesis. Since GABA is typically present at low levels in the human diet, foods enriched in this compound offer significant functional food and market potential. In addition, Suancai stored for one year showed markedly higher concentrations of valine and isoleucine than samples fermented for 180 days. These branched-chain amino acids serve as important precursors for the formation of malty, fruity, and sweet flavor compounds, thereby contributing to the sensory complexity of the product [34].

#### 3.2.3. Biogenic Amines

The biogenic amine contents of Suancai are presented in Figure 1D. During food fermentation and storage, biogenic amines are mainly generated through the decarboxylation of amino acids catalyzed by amino acid decarboxylases. In addition, certain aliphatic biogenic amines can be produced through the amination of aldehydes and ketones in combination with transamination reactions [35]. A total of seven biogenic amines were detected in both types of Suancai, namely, β-phenylethylamine, putrescine, cadaverine, tyramine, spermidine, spermine, and histamine, with overall concentrations ranging from 23.74 to 24.68 mg/kg. Both samples showed some accumulation of biogenic amines; however, the total levels remained relatively low due to the inherently low protein content of Suancai. Notably, cadaverine and histamine were absent in the 180-day fermented samples, while putrescine was undetectable in the one-year stored samples. Previous studies have reported putrescine as the predominant biogenic amine in northeastern Chinese Suancai. Although putrescine itself is not directly harmful to humans, it can enhance the intestinal absorption of histamine and tyramine, thereby exerting potential indirect health impacts [36]. Among biogenic amines, histamine and tyramine are considered the most hazardous to human health. In this study, their concentrations in both types of Suancai ranged from 2.37 to 5.21 mg/kg, which were relatively lower than the levels previously reported in Laotan Suancai fermented for 42 days [37].

### 3.3. Analysis of Volatile Compounds

#### 3.3.1. Qualitative Analysis of Volatile Flavor Compounds

**Figure 2:**
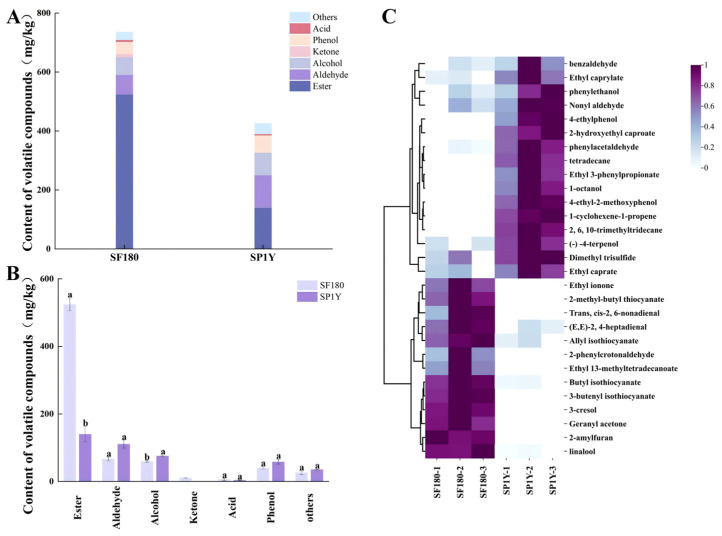
Volatile compounds in Suancai. (**A**) Total content of volatile compounds; (**B**) Classification of volatile compounds; (**C**) Clustering heatmap of differential volatile compounds screened by VIP values (>1.0). Different lowercase letters indicate differences in different Suancai (*p* < 0.05). SF180: Suancai fermented for 180 days. SP1Y: 1-year-aged Suancai post-fermentation. Data are presented on a fresh weight basis.

A total of 69 volatile compounds were detected across the two Suancai samples, with 51 compounds identified in the 180-day fermented sample and 49 in the one-year stored sample. These volatiles comprised 23 esters, 16 aldehydes, 8 alcohols, 3 ketones, 4 phenols, 2 acids, and 13 additional compounds (Table S1). The total volatile content measured 736.11 ± 20.38 mg/kg in the 180-day fermented Suancai but declined significantly to 426.42 ± 24.72 mg/kg after one year of storage (Figure 2A). Esters, alcohols, and aldehydes were the dominant categories in both samples, with esters being particularly abundant in the 180-day fermented Suancai, accounting for more than 50% of the total volatile compounds. However, after one year of storage, ester levels decreased markedly, while alcohol concentrations increased, as illustrated in Figure 2B. After one year of storage, the concentrations of key isothiocyanates, including allyl isothiocyanate, butyl isothiocyanate, and phenethyl isothiocyanate, decreased significantly (*p* < 0.05). This reduction can be attributed largely to the continued microbial metabolism of isothiocyanates during storage [38]. Since isothiocyanates are the primary degradation products of glucosinolates in mustard, the decline observed in this study is consistent with trends previously reported by Zhao et al. in other fermented vegetables [39]. The reduction of isothiocyanates weakened the characteristic pungent, garlic-like, and horseradish-like aroma of Suancai. From a consumer perspective, this shift in flavor profile may improve product acceptability by reducing sharp and overly intense sensory notes [40].

#### 3.3.2. Analysis of Differential Volatile Compounds

To further characterize differences in the volatile profiles of the two Suancai types, orthogonal partial least squares–discriminant analysis (OPLS-DA) was applied. This approach effectively distinguished variations in volatile composition between the samples and identified specific compounds that contributed most to the distinct flavor profiles. The OPLS-DA score plot (R^2^Y = 0.981, Q^2^ = 0.904) and the corresponding validation model (R^2^ = 0.724, Q^2^ = −0.642) are presented in Figure S1, confirming the robustness and reliability of the analysis. Based on variable importance in projection (VIP > 1.0) values from the OPLS-DA model, 29 volatile compounds were identified as significant contributors (Figure 2C). These included nine esters, seven aldehydes, four alcohols, three phenols, two ketones, and four additional compounds. As characteristic flavor markers distinguishing the two Suancai types, these volatiles play a pivotal role in defining their sensory profiles. The flavor quality of Suancai depends not only on the concentrations of volatile compounds but also on their OAVs, with higher OAVs indicating stronger contributions to overall aroma. Among the 29 compounds with VIP > 1.0, a total of 14 exhibited OAVs greater than 1 (Table 2), signifying their importance as key aroma-active components in shaping the distinctive flavor characteristics of Suancai.

As illustrated in Figure 2C, a cluster heatmap analysis was performed to visualize the composition and distribution of differential volatile compounds across Suancai samples. The analysis showed that Suancai fermented for 180 days contained 13 distinct volatile compounds, with ethyl ionone (OAV = 1,440,000, violet-like aroma), allyl isothiocyanate (OAV = 5483.7, mustard-like odor), and linalool (OAV = 3321.67, floral and woody notes) serving as representative contributors. In contrast, one-year stored Suancai was characterized by 16 volatile compounds that formed a separate cluster, representing its unique flavor profile. Among these, nonanal (OAV = 8436.36, sweet orange aroma), phenethylacetaldehyde (OAV = 7760, hyacinth-like notes), and ethyl decanoate (OAV = 614, brandy flavor) were identified as key aroma-active components (Figure 2C and Table 2). Aldehydes, mainly derived from amino acid and fatty acid metabolism, possess very low odor thresholds, allowing them to strongly influence sensory perception even at trace levels. Notably, linalool, a floral monoterpene synthesized through the methylerythritol phosphate pathway, emerged as the predominant alcohol in the 180-day fermented Suancai [41]. In contrast, phenethyl alcohol was identified as the predominant alcohol in Suancai stored for one year. This compound is derived from the catabolism of phenylalanine, and its accumulation corresponds with the reduced phenylalanine levels observed in the sample. Furthermore, previous studies have reported that the biosynthesis of phenethyl alcohol is strongly associated with the metabolic activity of *Lactobacillus* and *Weissella* [42].

### 3.4. Analysis of Microbial Diversity

#### 3.4.1. Succession of Bacterial and Fungal Communities

The species richness of Suancai was evaluated using the Observed Species and Chao1 indices, whereas microbial diversity was assessed using the Shannon and Simpson indices (Table S2). Analysis of these α-diversity metrics revealed clear differences in microbial community composition between the two sample types. Compared with the 180-day fermented Suancai, the one-year stored sample exhibited greater bacterial diversity, while fungal diversity remained relatively comparable between the two groups.

**Figure 3:**
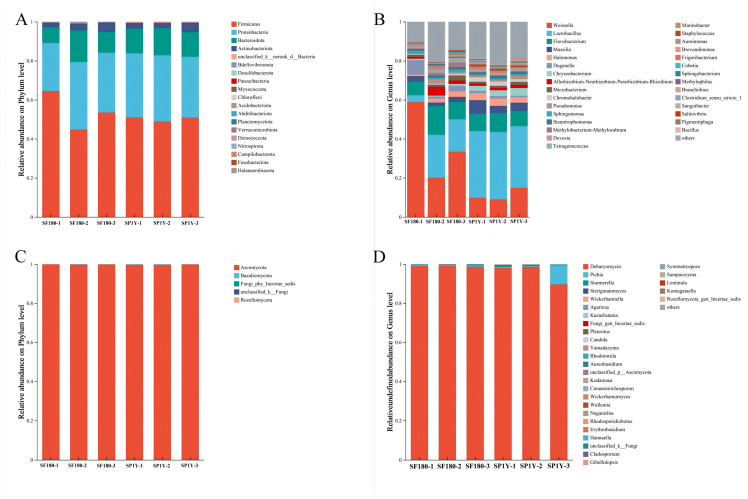
Dynamics of the microbial community in Suancai. (**A**) Bacterial genera; (**B**) bacterial phyla; (**C**) fungal genera; (**D**) fungal phyla. SF180: Suancai fermented for 180 days. SP1Y: 1-year-aged Suancai post-fermentation.

The microbial community composition of the two Suancai samples is presented in Figure 3. In terms of bacterial succession, members of the *Firmicutes* and *Proteobacteria* were predominant in both samples. These groups are well recognized as key contributors to the fermentation of Suancai, Paocai, and pickled bamboo shoots [43,44]. At the genus level, *Weissella* and *Lactobacillus* were the dominant taxa in both types of Suancai, a trend consistent with previous reports on the microbial ecology of other fermented vegetables, including radish Paocai, Chaozhou pickles, and Korean kimchi [45,46,47]. In Suancai fermented for 180 days, *Weissella* represented the largest proportion of the bacterial community (37.4%), followed by *Lactobacillus* (14%). However, after one year of storage, *Lactobacillus* became the dominant genus, exhibiting the highest relative abundance, while the proportion of *Weissella* declined to 11.2%, making it the second most prevalent genus. In addition, comparison of the two time points revealed a microbial shift characterized by an increase in the relative abundance of *Massilia* and a corresponding decrease in *Duganella*.

With respect to fungal succession, *Ascomycota* overwhelmingly dominated the fungal communities in both Suancai samples, accounting for more than 99% of the total relative abundance. At the genus level, *Debaryomyces* was the predominant taxon in both samples, representing 98.9% of the community in the 180-day fermented Suancai (SF180) and 95.5% in the one-year stored Suancai (SP1Y).

#### 3.4.2. Analysis of Species Differences

**Figure 4:**
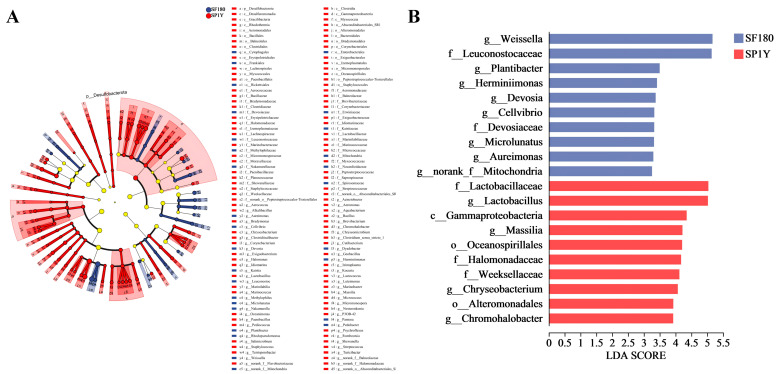
Linear discriminant analysis of the effect size (LEfSe) of microbial community. (**A**) Taxonomic cladograms of bacteria; (**B**) histogram of LDA score distribution of bacteria during fermentation. Labels beginning with p indicate phylum, c indicate class, o indicate order, f indicate family, and g indicate genus. SF180: Suancai fermented for 180 days. SP1Y: 1-year-aged Suancai post-fermentation.

The Linear Discriminant Analysis Effect Size (LEfSe) algorithm was applied to identify microbial taxa that significantly differentiated the two Suancai types. To ensure robust discrimination, the linear discriminant analysis (LDA) threshold was set at 3. An evolutionary cladogram of the discriminatory bacterial taxa identified by LDA is presented in Figure 4A. Comparative mapping against a taxonomically structured reference tree revealed 29 taxa with significantly different abundances in the 180-day fermented Suancai and 105 taxa with significant differences in the one-year stored Suancai (*p* < 0.05). As illustrated in Figure 4B, a total of 20 key microbial biomarkers were identified across the Suancai samples, including 1 class, 3 orders, 8 families, and 8 genera. These biomarkers represent consistent differential taxa distinguishing the 180-day fermented Suancai from the one-year stored samples. In the 180-day fermented Suancai, the dominant biomarkers included *Leuconostocaceae*, *Plantibacter*, *Herminiimonas*, and *Weissella*. By contrast, the one-year stored Suancai was primarily characterized by *Gammaproteobacteria*, *Lactobacillaceae*, *Lactobacillus*, and *Massilia*. Collectively, these findings demonstrate that the two Suancai types harbor distinct bacterial community structures, which are likely to play an important role in shaping their unique flavor characteristics [48].

### 3.5. Correlation Between Physicochemical Factors and Microorganisms

**Figure 5:**
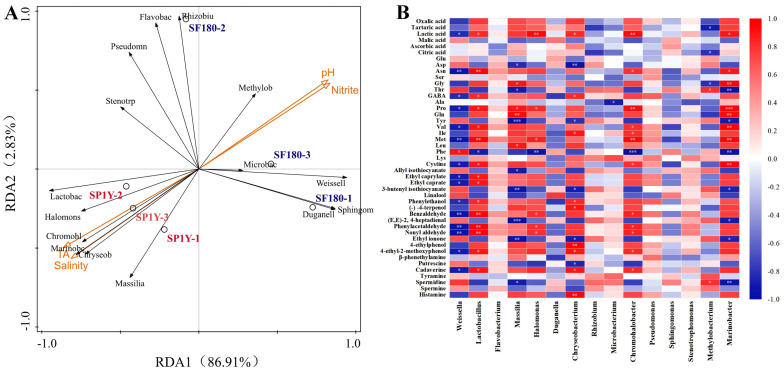
(**A**) Redundancy analysis (RDA) of microbial communities and physicochemical factors in Suancai. Weissell: Weissella; Lactobac: Lactobacillus; Flavobac: Flavobacterium; Halomons: Halomonas; Duganell: Duganella; Chryseob: Chryseobacterium; Rhizobiu: Rhizobium; Microbac: Microbacterium; Chromoha: Chromohalobacter; Pseudomn: Pseudomonas; Sphingom: Sphingomonas; Stenotrp: Stenotrophomonas; Methylob: Methylobacterium; Marinobac: Marinobacter. (**B**) Correlational analysis between microbial communities and metabolites. SF180: Suancai fermented for 180 days. *: *p* ≤ 0.05; **: *p *≤ 0.01; ***: *p* ≤ 0.001. SP1Y: 1-year-aged Suancai post-fermentation.

This study investigated the relationship between microbial community composition and physicochemical parameters using redundancy analysis (RDA) (Figure 5A). Axis 1 and Axis 2 together explained 86.91% and 2.83% of the total constrained variation, respectively. In the 180-day fermented Suancai, the microbial community was primarily characterized by the prevalence of *Weissella* and *Duganella*. In contrast, after one year of storage, *Lactobacillus*, *Halomonas*, and *Chromohalobacter* exerted a stronger influence on community structure. Among physicochemical variables, pH and nitrite content were positively associated with the 180-day fermented samples, whereas titratable acidity and salinity showed positive correlations with the one-year stored Suancai. A positive correlation was observed between *Lactobacillus*, *Chromohalobacter*, and *Marinobacter* and the levels of titratable acidity and salinity. In contrast, these genera were negatively correlated with pH and nitrite content. These findings are consistent with those reported by Wang et al. for northeastern sauerkraut [49]. Furthermore, previous studies have highlighted titratable acidity as one of a key factor driving microbial community succession in fermented foods [50].

### 3.6. Associations Among Microorganisms, Organic Acids, Free Amino Acids, Biogenic Amines, and Volatile Compounds

To explore the interrelationships among organic acids, free amino acids, biogenic amines, key flavor compounds, and core microbial communities in Suancai before and after storage, Spearman’s correlation analysis was conducted. Fourteen volatile compounds with both VIP values and OAVs > 1 were identified as key flavor compounds. Meanwhile, bacterial and fungal genera with relative abundances exceeding 1% were designated as the core microbiota.

As shown in Figure 5B, *Lactobacillus*, *Halomonas*, and *Chromohalobacter* exhibited strong positive correlations with lactic acid, while *Weissella* was significantly negatively correlated with lactic acid levels (*p* < 0.05). This finding is consistent with earlier studies reporting the central role of *Lactobacillus* in lactic acid production during fermentation [51]. In addition, *Lactobacillus*, *Chromohalobacter*, and *Marinobacter* showed significant positive correlations with proline, valine, and methionine, whereas *Weissella* displayed negative correlations with these amino acids (*p* < 0.05). Regarding key flavor compounds, *Lactobacillus*, *Halomonas*, and *Chromohalobacter* were strongly positively associated with benzaldehyde, phenethylaldehyde, and nonanal, indicating their possible roles in shaping the aroma profile of Suancai. Previous studies have shown that *Lactobacillus* contributes to the biosynthesis of benzaldehyde and phenethylaldehyde [52]. Consistent with this, in the present study, *Lactobacillus* exhibited strong positive correlations with the esters ethyl octanoate and ethyl decanoate, whereas *Weissella* was negatively correlated with these compounds (*p* < 0.05). *Debaryomyces* also displayed significant positive correlations with aspartic acid, tyrosine, allyl isothiocyanate, 3-butenyl isothiocyanate, and ethyl ionone, but negative correlations with isoleucine and leucine (*p* < 0.05). Collectively, these results highlight the substantial influence of both bacterial and fungal genera on amino acid metabolism, ester and isothiocyanate production, and ultimately on the development of taste and flavor characteristics in Suancai.

## 4. Conclusions

This study reveals that the storage period serves as a critical phase driving substantial shifts in the microbial ecology and metabolite profiles of Suancai, which collectively govern the final product quality, based on a systematic investigation of samples at the end of fermentation and after one-year storage. After one year of storage, the nitrite content in Suancai was significantly reduced, while the γ-aminobutyric acid content was significantly increased, jointly enhancing the safety and nutritional value of the product. Post-storage Suancai underwent a fundamental flavor transition from a pungent, floral profile to a matured, fruity-floral character. This shift was driven by a marked turnover in volatile compounds, characterized by a decline in esters and pungent isothiocyanates alongside an increase in alcohols and key aldehydes. Concurrently, the significant accumulation of non-volatile metabolites, including specific organic acids and amino acids, indicates that microbial metabolic activity persists throughout storage, continuously shaping the product’s taste foundation. At the microbial level, *Lactobacillus* supplanted *Weissella* as the dominant genus, with significant correlations observed between *Lactobacillus* and lactic acid, sweet amino acids, and key flavor compounds (e.g., ethyl decanoate). These associations underscore the central regulatory role of core microbiota in shaping metabolic profiles and quality attributes. Additionally, the positive link between *Debaryomyces* and isothiocyanates highlights the specific contribution of fungal taxa to the flavor characteristics of Suancai. This research reveals that the storage period plays a definitive role in governing end product quality through dynamic microbe–metabolite interactions, providing a new perspective for predicting and controlling flavor during shelf life.

As this study compared only two time points (pre- and post-storage), it could not reveal the dynamic changes in the microbiota and metabolites throughout the entire storage period. Thus, future work with more frequent sampling and metatranscriptomics is needed to pinpoint the critical drivers of flavor transition.

## Figures and Tables

**Table 1:** Physicochemical Properties of both Suancai Types.

	pH	Titratable Acidity (g/L)	Salinity (%)	Nitrite (mg/kg)
Suancai fermented for 180 days	4.88 ± 0.02 ^a^	3.35 ± 0.31 ^b^	16.10 ± 0.10 ^b^	0.67 ± 0.00 ^a^
1-year-aged Suancai post-fermentation	3.62 ± 0.02 ^b^	7.54 ± 0.15 ^a^	18.90 ± 0.10 ^a^	0.09 ± 0.00 ^b^

Different superscript letters within the same column indicate statistically significant differences between groups (*p* < 0.05). Data are expressed as the mean ± standard deviation. Data are presented on a fresh weight basis.

**Table 2:** Volatile Compounds in Suancai with both VIP and OAV Values > 1 (n = 14).

No.	Compounds	CAS	OT ^1^ (mg/kg)	Odor Description ^2^	OAVs	
					Suancai Fermented for 180 Days	1-Year-Aged Suancai Post-Fermentation
	**Esters**					
1	Allyl isothiocyanate	57-06-7	0.046	Pungent mustard smell	5483.70	1390.87
2	Ethyl caprylate	106-32-1	0.82	Brandy’s fragrant sweetness	3.24	6.29
3	Ethyl caprate	110-38-3	0.005	Fruity aroma, brandy-like aroma	174	614
4	3-butenyl isothiocyanate	3386-97-8	0.017	Strong, pungent smell reminiscent of rubber	2325.29	/
	**Alcohols**					
5	Linalool	78-70-6	0.006	Sweet, typical floral	3321.67	353.33
6	Phenethyl alcohol	60-12-8	0.564	Floral scent	40.09	74.10
7	(-)-4-terpenol	20126-76-5	0.04	Peppery, woody	15.75	114.25
	**Aldehydes**					
8	Benzaldehyde	100-52-7	0.35	Bitter almond scent	34.31	58.4
9	(E,E)-2,4-heptadienal	4313-03-5	0.0154	fatty	881.82	203.90
10	phenylacetaldehyde	122-78-1	0.004	Hyacinth fragrance	2035	7760
11	Nonyl aldehyde	124-19-6	0.0011	Strong fatty odor, sweet orange scent.	5554.54	8436.36
	**Ketones**					
12	Ethyl ionone	79-77-6	0.000007	Violet fragrance	1,440,000	/
	**Phenols**					
13	4-ethylphenol	123-07-9	0.013	castoreum	/	1070
14	4-ethyl-2-methoxyphenol	2785-89-9	0.05	Herbaceous fragrance	/	836.6

^1^ Odour thresholds in water. ^2^ odour descriptions sourced from https://pubchem.ncbi.nlm.nih.gov/ (accessed on 15 October 2024) and http://www.flavornet.org/ (accessed on 15 October 2024). “/” means not detected. Data are presented on a fresh weight basis.

## Data Availability

The original contributions presented in this study are included in the article/Supplementary Materials. Further inquiries can be directed to the corresponding author.

## Supplementary Materials

The following supporting information can be downloaded at: https://www.mdpi.com/article/10.3390/foods14203490/s1, Table S1. Volatile compounds in Suancai; Table S2. Alpha diversity indices of the Suancai microbial community; Figure S1. The orthogonal partial least squares discrimination analysis (OPLS-DA) plot and cross-validation of Suancai.
