# Supplementary material for: Alterations in Biochemical Characteristics, Flavor, and Microbial Community During the Storage of Suancai
Source: Foods. 2025 Oct 14;14(20):3490. doi: 10.3390/foods14203490 (PMC12563257; doi:10.3390/foods14203490)
Supplement: Supplementary file 1 [file foods-14-03490-s001.zip › foods-3890192-supplementary.pdf]

Table S1. Volatile compounds in Suancai.

No.	Compounds	CAS	Odor description	Concentration (mg/kg）	
				Suancai fermented for 180 days	1-year-aged Suancai post-fermentation
	**Esters**				
1	Allyl isothiocyanate	57-06-7	Pungent mustard smell	277.79 ±8.88 [a]	51.25±16.54 [b]
2	N-hydroxybenzoimide methyl ester	67160-14-9	/	4.80 ±6.79 [a]	1.5±2.12 [a]
3	Butyl isothiocyanate	591-82-2	the pungent smell of oranges/citrus	42.70±1.29 [a]	4.05±0.86 [a]
4	Ethyl caprylate	106-32-1	Brandy's fragrant sweetness	2.64±0.36 [b]	4.61±0.13 [a]
5	Ethyl caprate	110-38-3	Fruity aroma, brandy-like aroma	0.76±1.07 [a]	2.63 ±0.53[a]
6	Phenyl ethyl isothiocyanate	2257-09-2	Mustard-like smell	32.37±0.42 [a]	1.65±2.33 [b]
7	Ethyl laurate	106-33-2	Fruity aroma	0.78±1.10 [a]	1.18±0.81 [a]
8	Octyl salicylate	118-60-5	Slightly fragrant	28.77±8.09 [a]	18.97±10.22 [a]
9	Isopropyl myristate	110-27-0	/	1.84±0.96 [a]	0.48±0.68 [a]
10	Dodecyl isoamyl ester	6309-51-9	Faint oil and ester fragrance	5.97±2.11 [a]	4.33 ±2.31 [a]
11	Diisobutyl phthalate	84-69-5	fragrant smell	0.69±0.09 [a]	0.52±0.01 [a]
12	3,3, 5-trimethylcyclohexanol salicylate	118-56-9	/	26.28±6.76 [a]	16.36±9.31 [a]
13	Ethyl pentadecanoate	41114-00-5	Honey sweetness	0.71±1.00 [a]	0.33±0.46 [a]
14	Methyl palmitate	112-39-0	Oily, waxy iris scent	6.03±1.62 [a]	3.09±1.55 [a]
15	Ethyl palmitate	628-97-7	Fruity and creamy flavor	19.16±4.71[a]	10.22±4.88 [a]
16	Isopropyl palmitate	142-91-6	/	12.38±3.07 [a]	5.19±3.04 [a]
17	3-butenyl isothiocyanate	3386-97-8	Strong pungent smell reminiscent of rubber	42.11±0.47	-
18	2-methyl-butyl thiocyanate	4404-51-7	Strong pungent odor	16.52±1.61	-
19	Ethyl 13-methyltetradecanoate	64317-63-1	/	2.17±0.86	-
20	2-hydroxyethyl caproate	/	/	-	8.86±2.68
21	Ethyl enanthate	106-30-9	Pineapple fragrance	-	0.38±0.53
22	Phenyl ethyl acetate	103-45-7	floral rose sweet honey fruity tropical	-	1.67±2.36
23	Ethyl 3-phenylpropionate	2021-28-5	hyacinth rose honey fruity	-	2.92±0.81
	**Alcohols**				
24	linalool	78-70-6	Sweet, typical, floral and woody fragrance	20.25±1.63 [a]	2.18±0.28 [b]
25	phenylethanol	60-12-8	Floral scent	24.75±3.82 [b]	48.79±4.64 [a]
26	E-11, 13-tetradecene-1-ol	/	/	13.11±1.05 [a]	16.21±1.20 [a]

27	(-) -4-terpenol	20126-76-5	Peppery woody blend	0.45±0.64 [b]	4.88±0.78 [a]
28	cyclooctanol	696-71-9	/	0.26±0.37	-
29	1-pentadecanol	629-76-5	Subtle, mild, waxy, and faint floral notes	1.14±0.33	-
30	1-octanol	111-87-5	Strong oily odor	-	3.33±0.37
31	DL-beta-ethylphenylethylethanol	2035-94-1	Aromatic fragrance	-	0.95±0.13
	**Aldehydes**				
32	benzaldehyde	100-52-7	Bitter almond scent	12.82±0.92 [a]	23.31±5.72 [a]
33	(E,E)-2, 4-heptadienal	4313-03-5	fatty	15.23±0.40 [a]	3.82±0.95 [b]
34	phenylacetaldehyde	122-78-1	Hyacinth fragrance	8.53±1.00 [b]	33.71±3.13 [a]
35	Nonyl aldehyde	124-19-6	Strong fatty odor, sweet orange scent.	6.65±0.86[a]	10.27±0.06[a]
36	Capric aldehyde	112-31-2	Pleasant aroma	9.62±1.64 [a]	12.66±1.35 [a]
37	beta-cyclocitral	432-25-7	Herbal	8.17±0.45 [a]	5.74±0.55 [b]
38	2-heptenal	2463-63-0	/	0.51±0.71	-
39	Trans, cis-2, 6-nonadienal	557-48-2	Violet leaf scent, cucumber aroma	2.58±0.04	-
40	2-phenylcrotonaldehyde	4411-89-6	Moldy fragrance, floral aroma	2.04±0.91	-
41	5, 9, 13-trimethyl-4, 8, 12-tetradecatrienal	/	/	1.09±0.20	-
42	(E)-Hept-2-enal	18829-55-5	Spicy, leafy greens	-	1.11±0.51
43	caprylaldehyde	124-13-0	Fruity fragrance	-	4.50±0.07
44	trans-2-octenal	2548-87-0	Fatty and meaty aroma	-	3.91±0.00
45	(Z) -2-nonenal	60784-31-8	/	-	1.17±0.21
46	2, 3-dihydro-2,2, 6-trimethylbenzaldehyde	116-26-7	Strong fragrance	-	0.69±0.98
47	1-cyclohexene-1-propene	/	/	-	10.29±0.29
	**Ketones**				
48	Geranyl acetone	689-67-8	Magnolia fragrance	2.06±0.14	-
49	Ethyl ionone	79-77-6	Violet fragrance	8.62±1.21	-
50	phytoketone	502-69-2	oily herbal jasmin celery	0.35±0.49	-
	**Phenols**				
51	2, 4-di-tert-butylphenol	96-76-4	/	38.13±3.08 [a]	7.14 ±10.09 [a]
52	3-cresol	108-39-4	Phenolic odor	2.55±0.26	-
53	4-ethylphenol	123-07-9	castoreum	-	12.68±6.41
54	4-ethyl-2-methoxyphenol	2785-89-9	Herbaceous fragrance	-	38.67±11.05
	**Acids**				
55	7, 10, 13-hexadecatrienoic acid	/	/	6.41±3.46	
56	Caproic acid	142-62-1	fatty	-	4.50±1.00
	**Others**				
57	Dimethyl trisulfide	3658-80-8	Intense spicy aroma	4.31±3.83 [b]	14.10±0.04 [a]
58	Methylallyl trisulfide	34135-85-8	Garlic flavor	1.98±0.06 [b]	2.78±0.04 [a]

59	Phenylpropionitrile	645-59-0	/	8.8±1.46 [a]	11.26±0.01 [a]
60	Nonadecane	629-92-5	/	1.31±0.32 [a]	1.74±0.21 [a]
61	1,8,11, 14-heptadecatetraene	/	/	2.16±0.25	-
62	Edwardian II	41678-30-2	/	1.85±1.32	-
63	2H-1b, 4-ethanopentene [1, 2-b]	/	/	2.20±1.56	-
64	Eicosane	112-95-8	Odorless	0.16±0.22	-
65	2-amylfuran	3777-69-3	Fruity, Green	3.33±0.17	-
66	Trans-2 -(2-pentenyl) furan	/	/	3.59±2.24	-
67	1-methoxy1h indole	54698-11-2	/	-	1.35±0.45
68	tetradecane	629-59-4	Mild waxy flavor	-	0.84±0.13
69	2, 6, 10-trimethyltridecane	3891-99-4	/	-	2.41±0.18

Odor descriptions sourced from https://pubchem.ncbi.nlm.nih.gov/ and http://www.flavornet.org/. "/"means no references are available. "-" means not detected. Data are presented on a fresh weight basis.

Table S2. Alpha diversity indices of the Suancai microbial community.

Sample	Sobs	Chao	Shannon	Simpson
**Bacteria**				
SF180-1	627	635.28	3.32	0.18
SF180-2	758	758.00	4.29	0.05
SF180-3	746	748.43	4.47	0.04
SP1Y-1	944	946.54	4.84	0.02
SP1Y-2	900	907.29	4.71	0.02
SP1Y-3	941	956.25	4.88	0.02
Fungi				
SF180-1	21	21	0.10	0.97
SF180-2	22	24.5	0.07	0.98
SF180-3	22	23.2	0.10	0.97
SP1Y-1	21	21	0.14	0.96
SP1Y-2	30	31	0.11	0.97
SP1Y-3	18	18	0.42	0.81

SF180: Suancai fermented for 180 days. SP1Y: 1-year-aged Suancai post-fermentation.

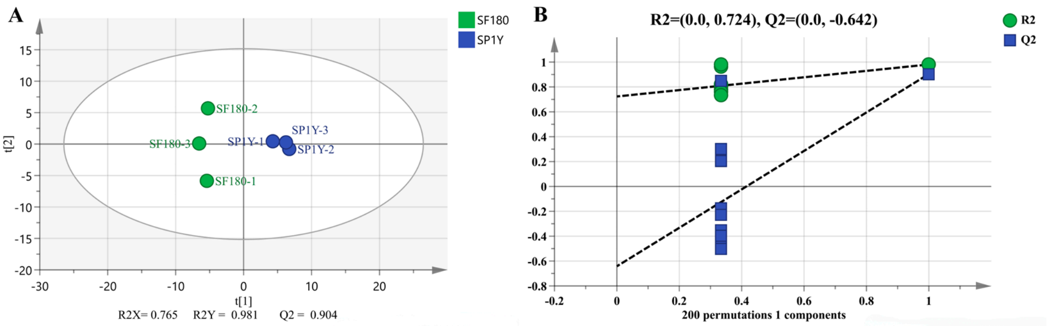

Figure S1. The orthogonal partial least squares discrimination analysis (OPLS-DA) plot and cross-validation of Suancai. (**A**) Score plot of OPLS-DA ($R^2Y = 0.981$, $Q^2 = 0.904$). (**B**) Validation model of OPLS-DA with 200 permutation tests ($R^2 = 0.724$, $Q^2 = -0.642$). SF180: Suancai fermented for 180 days. SP1Y: 1-year-aged Suancai post-fermentation.
